# Cdk12 Is A Gene-Selective RNA Polymerase II Kinase That Regulates a Subset of the Transcriptome, Including Nrf2 Target Genes

**DOI:** 10.1038/srep21455

**Published:** 2016-02-25

**Authors:** Xuan Li, Nirmalya Chatterjee, Kerstin Spirohn, Michael Boutros, Dirk Bohmann

**Affiliations:** 1 Department of Biomedical Genetics, University of Rochester Medical Center, Rochester, NY 14642, USA; 2 Division of Signaling and Functional Genomics, German Cancer Research Center (DKFZ) and Department of Cell and Molecular Biology, Medical Faculty Mannheim, Heidelberg University, Heidelberg, Germany

## Abstract

The Nrf2 transcription factor is well conserved throughout metazoan evolution and serves as a central regulator of adaptive cellular responses to oxidative stress. We carried out an RNAi screen in *Drosophila* S2 cells to better understand the regulatory mechanisms governing Nrf2 target gene expression. This paper describes the identification and characterization of the RNA polymerase II (Pol II) kinase Cdk12 as a factor that is required for Nrf2 target gene expression in cell culture and *in vivo*. Cdk12 is, however, not essential for bulk mRNA transcription and cells lacking CDK12 function are viable and able to proliferate. Consistent with previous findings on the DNA damage and heat shock responses, it emerges that Cdk12 may be specifically required for stress activated gene expression. Transcriptome analysis revealed that antioxidant gene expression is compromised in flies with reduced Cdk12 function, which makes them oxidative stress sensitive. In addition to supporting Reactive Oxygen Species (ROS) induced gene activation, Cdk12 suppresses genes that support metabolic functions in stressed conditions. We suggest that Cdk12 acts as a gene-selective Pol II kinase that engages a global shift in gene expression to switch cells from a metabolically active state to “stress-defence mode” when challenged by external stress.

The transcription factor Nrf2 plays critical roles in cellular responses to exogenous and endogenous stress and protects organisms from oxidative damage^1^,^2^. Nrf2 target genes encode proteins that safeguard cellular homeostasis, including antioxidants and detoxification factors. These genes become activated when cells are confronted with potentially threatening levels of stress^3^. Nrf2 controls the expression of its downstream target genes by binding to specific promoter sequence called the Antioxidant Response Element (ARE)^4^. Consistent with its stress-defence function, Nrf2 is critical for protecting organisms from oxidative stress-related pathologies, such as cancer, inflammatory and neurodegenerative diseases^5^,^6^.

Several signalling pathways that can regulate Nrf2 activity have been described. Best known is the interaction between Nrf2 and its inhibitor Keap1, which mediates cullin 3-dependent proteasomal degradation of Nrf2. The binding of Keap1 to Nrf2 is released in oxidative stress conditions causing stabilisation and nuclear translocation of Nrf2^7^,^8^. Nrf2 can also be regulated in a Keap1-independent manner via GSK-3, which phosphorylates Nrf2 and promotes its degradation by a mechanism involving the ubiquitin ligase adapter β-TrCP and cullin 1^9^. The inhibition of Nrf2 by GSK-3 phosphorylation can be controlled by PI3K and Akt signalling, accounting for effects of growth factors and metabolic signals on Nrf2 function^10^. While our knowledge of the signal-dependent regulation of Nrf2 target gene activation is expanding, it is still incomplete. In this paper we describe studies of Nrf2 regulation using the model organism *Drosophila* and the powerful genetic and genomic tools that are available in this system. There is remarkable conservation of Nrf2 signalling between *Drosophila* and mammals. The Nrf2 homolog in *Drosophila* is the CncC protein, one of several gene products that are generated from the *cnc* (cap’n’collar) locus by alternative splicing^5^,^11^,^12^.

In an effort to better understand the regulatory mechanisms controlling Nrf2, we carried out an unbiased RNAi screen to identify novel regulators of CncC pathway activity. The screen found Cdk12, a Pol II kinase^13^, to be specifically required for efficient CncC target gene activation. Our results corroborate and extend recent findings which suggest that Cdk12 acts as a gene-selective Pol II kinase with specialized functions in different types of cellular stress responses^13^,^14^.

## Results

### An RNAi screen for mediators of Nrf2 target gene activation identifies Cdk12, an RNA polymerase II kinase

**Figure 1:**
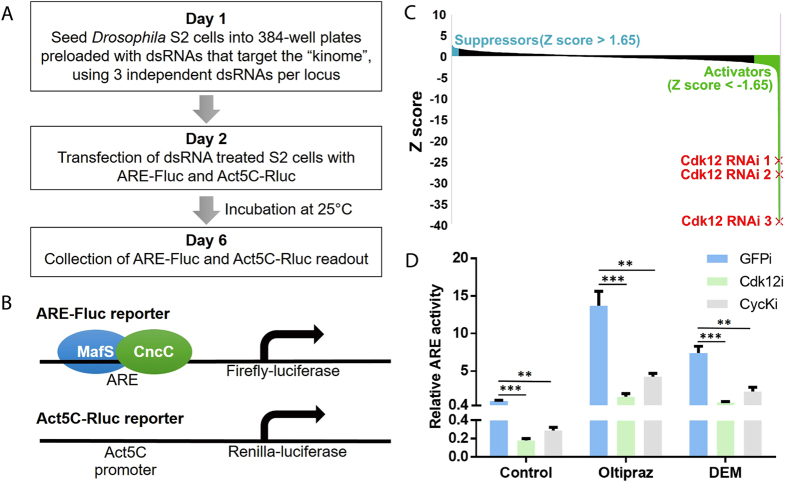
A kinome-wide RNAi screen identifies Cdk12 as a positive regulator of CncC target gene activity. (**A**) Flowchart describing the RNAi screening strategy to identify genes affecting CncC target gene activation. Fluc: firefly luciferase; Rluc: *Renilla* luciferase. (**B**) Schematic representations of two reporters used in the RNAi screen. MafS is the dimerization partner of CncC during the transcriptional activation of CnCC target genes. (**C**) dsRNAs that cause a decrease or an increase in reporter gene activity, respectively, identify putative activators (highlighted in blue) or suppressors (highlighted in green) of CncC-dependent transcription. The strongest activator identified in the screen was Cdk12. (**D**) Knockdown of Cdk12 in *Drosophila* S2 cells leads to a significant decrease in ARE-Fluc reporter activity under basal, as well as oltipraz- or DEM-treated conditions. Knockdown of cyclin K, a regulatory subunit interacting with Cdk12, causes a similar decrease in ARE-Fluc signal to knockdown of Cdk12 itself. ARE-Fluc reporter readout was normalized to renilla luciferase activity from the internal control plasmid Act5C-Rluc. Normalized ARE-Fluc activity is expressed relative to its activity under control condition, which was set to 1 (no inducer, GFP dsRNA treated). P values were calculated using multiple t-tests; **P < 0.005, ***P < 0.0005. Error bars represent standard deviations of 3 biological replicates.

We designed a cell-based RNAi screen to identify factors that affect transcription regulation by the *Drosophila* Nrf2 homolog, CncC (Fig. 1A). Kinase-dependent mechanisms have been implicated in Nrf2 regulation but have not been exhaustively characterized^15^,^16^,^17^,^18^,^19^,^20^,^21^,^22^. Accordingly, we chose to focus our screen specifically on kinases and employed an RNAi library targeting all known or putative kinases in the *Drosophila* genome (the “kinome”). We used a cell culture-based transcriptional reporter system that we had previously developed^23^. Nrf2-responsive ARE-firefly-luciferase (ARE-Fluc) and Act5C-renilla-luciferase (Act5C-Rluc) plasmid constructs served as reporters for CncC transcriptional activity and internal control, respectively (Fig. 1B). Employing a robotic 384-well platform and dsRNA-mediated gene knockdown, we conducted a screen to identify kinases that might affect reporter gene activity in transiently transfected *Drosophila* Schneider 2 (S2) cells. Under the conditions of the screen CncC was moderately active so that both activating and repressive effects could be discerned. In control experiments, knockdown of CncC itself, or its required dimerization partner MafS caused a significant decrease in ARE-Fluc activity. Conversely, an increased signal was observed after knockdown of the CncC inhibitor Keap1 (Table 1). These validation experiments confirmed that the assay on which the screen is based can be used to quantitatively assess Nrf2 activity.

432 genes encoding presumptive kinases were tested in the screen, using typically three independent dsRNAs per locus. Each of the three dsRNAs targeted different parts of an mRNA to exclude the possibility of off-target effects. Genes that upon knockdown caused a significant decrease in Nrf2 reporter activity for all three dsRNAs (Z-scores below −1.65) were classified as potential positive regulators. Conversely, cases where knockdown caused a consistent increase in luciferase activity (Z-scores above +1.65) identified putative Nrf2 inhibitors (Fig. 1C and Table 1). Among the genes that fulfil these criteria we found a number of plausible hits which had previously been implicated in Nrf2 function or longevity regulation. Examples include GSK-3 and CK2^10^,^16^,^18^,^24^,^25^. In addition, a number of other less well characterized kinases as well as kinases without known connections to Nrf2 or aging also scored in the assay. Among these was Cdk12, a Pol II CTD kinase. Out of all tested genes, knockdown of Cdk12 had the biggest repressive effect on ARE luciferase activity, making this kinase the most potent positively functioning factor identified in the screen. Cdk12 is one of the kinases that phosphorylate serine residues in the C-terminal domain (CTD) of the largest subunit of Pol II^13^,^14^. In *Drosophila* the CTD is composed of multiple repeats of the sequence YSPTSPS^26^. Phosphorylation of serine residues in these repeats plays critical roles in the initiation and productive elongation of mRNA transcription, as well as several steps of mRNA processing, including 5′ capping, splicing and 3′ end formation^27^,^28^. CDK12 in complex with a dedicated regulatory subunit, Cyclin K (CycK), can specifically phosphorylate the Ser2 residue in the CTD repeat sequence^14^,^29^,^30^,^31^. For every protein-coding gene, the phosphorylation of the Ser2 residues in the Pol II CTD region is essential for the transition from transcription initiation to elongation and thus the synthesis of full-length mRNA transcripts^27^,^32^. Cdk12 is one of two *Drosophila* enzymes with this substrate specificity, the other one being Cdk9, which forms a functional complex with Cyclin T (CycT)^13^.

Next, we validated the RNAi screen results and examined whether CDK12 is important for the reporter gene response to upstream signals that activate Nrf2. For these experiments we employed the selective Nrf2 activator oltipraz and the oxidizing agent diethyl maleate (DEM). Oltipraz acts by interfering with the Nrf2 inhibitor Keap1, thereby stabilizing the transcription factor without causing harmful levels of cell stress^33^. DEM activates the Nrf2 signalling pathway by depleting intracellular glutathione and causing oxidative stress^34^. ARE-luciferase assays in *Drosophila* S2 cells showed that Cdk12 knockdown led to a substantial decrease in ARE-Fluc signal in the absence or presence of oltipraz and DEM, arguing that Cdk12 is required for the activity of Nrf2 pathway under standard S2 cell culture conditions and after extracellular stimulation (Fig. 1D). Notably, knockdown of CycK, the cyclin partner of Cdk12, decreased Nrf2 pathway activity to a similar extent as knockdown of Cdk12 itself in control, oltipraz and DEM treated conditions (Fig. 1D). This result demonstrated that CycK is required as the binding and functional partner of Cdk12 in mediating Nrf2-dependent gene regulation.

Cdk12 acts on the Ser2 residue in Pol II CTD heptad repeats, the phosphorylation of which is essential for the expression of Pol II-transcribed genes^27^,^32^. Interestingly however, while a >85% knockdown of Cdk12 in S2 cells strongly suppressed Nrf2 reporter gene activity, it did not affect cell viability, nor did it cause a marked change in the activity of the internal control construct, the renilla luciferase reporter that is driven by an Act5C promoter. This result implied that the activity of Cdk12 may not be universally required for all Pol II-dependent transcription, especially of essential housekeeping genes. Rather, it may only support transcription of a subset of genes, which are apparently dispensable for cell viability under standard cell culture conditions. This subset presumably includes CncC-regulated genes. Consistent with this interpretation, some recent papers described a possible gene selective function of Cdk12 and implicated the kinase in the transcriptional control of heat shock and DNA damage response genes^13^,^14^. From these studies and the findings presented here, it emerges that the specificity of transcription regulation might not only be mediated at the level of sequence-specific DNA-binding transcription factors, but also potentially at the level of Pol II phosphorylation through the utilization of different CTD kinases.

### Cdk12 is required for Nrf2 reporter activity *in vivo*

**Figure 2:**
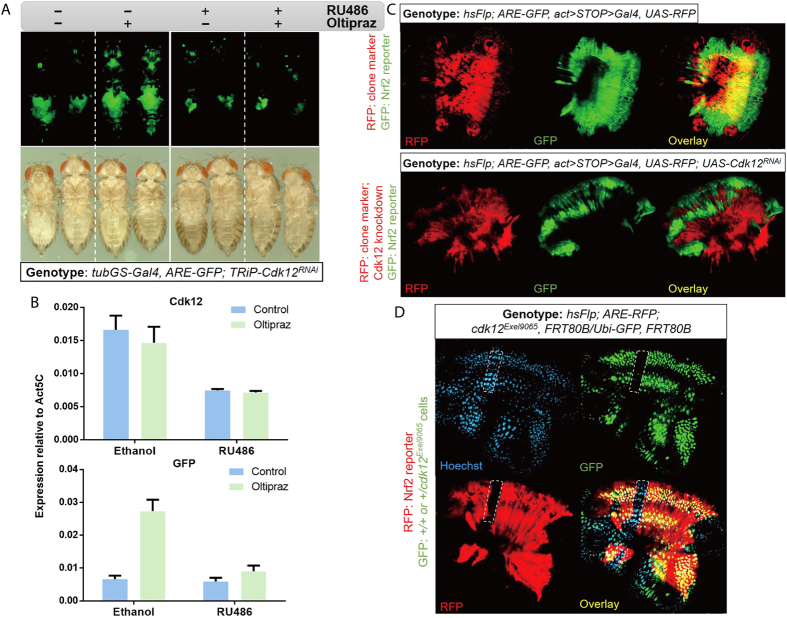
Cdk12 supports CncC target gene expression *in vivo*. (**A**) Knockdown of Cdk12 by expression of an RNAi construct under the control of the ubiquitously active gene-switch tub-Gal4 driver prevents oltipraz-induced ARE-GFP reporter activation in adult flies. The upper and lower panels show GFP fluorescence and bright field images, respectively, of the same flies. Two females are shown for each condition. (**B**) RT-qPCR quantification of Cdk12 and GFP mRNA in flies of the indicated treatment groups. The genotype of the flies is tubGS-Gal4, ARE-GRP; UAS-Cdk12^RNAi^. mRNA expression levels were normalized to Act5C. Error bars indicate standard deviations of 3 biological replicates. Each replicate included a population of 10 flies. (**C**) Cdk12^RNAi^-expressing clones in the ejaculatory bulb display low Nrf2 reporter activity. The upper and lower panels represent control and Cdk12^RNAi^-expressing clones, respectively. RFP, GFP channels and the overlay of both are shown. (**D**) A clone of ejaculatory bulb cells that are homozygous for the null allele *cdk12*^*Exel9065*^ is viable and able to grow but lacks Nrf2 reporter activity. The Cdk12 mutant clone, which is marked by the absence of GFP and outlined by a dashed white line, displays low levels of ARE-RFP reporter signal. DAPI, GFP, RFP channels and the overlay of all these channels are shown individually.

After the initial identification and characterization of Cdk12 as a factor contributing to CncC-dependent gene activation in S2 cells, we analysed the function of this CTD kinase *in vivo*. To this end, we tested the effect of Cdk12 loss-of-function on an *in vivo* ARE-GFP reporter that we have previously described^23^ in *Drosophila melanogaster*. We used a transgenic construct expressing a hairpin RNA that can be processed into Cdk12-specific RNAi. This transgene was expressed under the control of a ubiquitously active RU486-inducible Gal4 driver in adult flies carrying the ARE-GFP reporter. In control flies where no RNAi was expressed, oltipraz treatment robustly activated the GFP signal, especially in the thorax and abdominal regions, as assessed by inspection of whole flies under fluorescent microscopy. In contrast, ubiquitous Cdk12 knockdown induced by RU486 treatment abolished the upregulation of the ARE-GFP reporter signal almost completely, and flies with and without oltipraz treatment displayed basically indistinguishable low GFP activity (Fig. 2A). This indicated that Cdk12 is critical for the signal-dependent activation of Nrf2 pathway also in the intact organism. Of note, this result recapitulated the effect of knocking down CncC itself (Supplementary Fig. S1). The effect of Cdk12 knockdown on Nrf2 reporter activation was reproduced with an independent fly line that expresses a hairpin RNA targeted to a different, non-overlapping region of Cdk12 mRNA (Supplementary Fig. S2). This result excluded the possibility of off-target effects and confirmed the specificity of Cdk12 knockdown. For quantification RNA from pooled flies of the relevant genotype and treatment groups was extracted and the relative expression level of GFP was assessed by RT-qPCR. The results confirmed the conclusions of the qualitative fluorescence microscopy experiment, showing a decrease in the fold induction of GFP mRNA abundance from 4.1 to 1.5 fold. Efficient knockdown of Cdk12 was also achieved as evidenced by the qPCR data (Fig. 2B). Altogether these results argue that Cdk12 is required for the activation of Nrf2 reporter activity *in vivo*.

In the experiments described above, Cdk12 function was manipulated organism-wide by RNAi constructs that were expressed under the control of the ubiquitously active tubulin promoter. Next, we tested the effect of Cdk12 loss at the tissue and cell level. To this end, we employed an Flp-recombinase-FRT strategy to express the Cdk12 RNAi construct in individual, genetically marked clones. Clones were generated by heat shock-induced expression of Flp recombinase, causing the heritable activation of Gal4 expression. The Gal4/UAS system in turn activated the RNAi transgene as well as a transgene expressing RFP, which served as a marker (Fig. 2C). We chose the ejaculatory bulb for this analysis, because it displays a high endogenous level of Nrf2 activity, while this activity is low in most other fly tissues at the basal level. In control clones where Cdk12 RNAi was not expressed, the ARE-GFP reporter signal remained indistinguishable from that of the neighbouring cells (Fig. 2C upper panel). In contrast, in clones expressing Cdk12 RNAi, the GFP signal disappeared (Fig. 2C lower panel). The elimination of the ARE-GFP signal was seen strictly within the clonal boundaries, but not in surrounding cells where Cdk12^RNAi^ construct was not expressed, indicating that the effect of Cdk12 is cell autonomous and confirming the requirement of Cdk12 for sustaining the activity of Nrf2 signalling pathway. Consistent with the cell culture assays, in the above experiments all Cdk12 knockdown flies and clones were viable and the clones were morphologically indistinguishable from neighbouring wild type cells. This suggests that wild type levels of Cdk12 expression are not required for general Pol II-dependent RNA transcription, which is essential for cell growth and viability. This finding supports the notion that Cdk12 is not required for housekeeping functions, but that it may have a more specialized regulatory role.

Besides knocking down Cdk12 in individual clones using an RNAi strategy, we also examined the effect of a Cdk12 null allele on Nrf2 reporter activity and cell viability in mutant clones of the ejaculatory bulb tissue. In the *cdk12*^*Exel9065*^ allele, the open reading frame of Cdk12 is deleted. This allele is homozygous lethal, possibly because Cdk12 function is needed for successful development. Cdk12 deficient clones were generated by mitotic recombination of FRT chromosomes carrying the *cdk12*^*Exel9065*^ allele and marked by the lack of GFP fluorescence (Fig. 2D). Consistent with results of the knock down experiments (Fig. 2C), the chromosomal Cdk12 deletion abrogated the ARE-RFP reporter activity. However, Cdk12 mutant cells survived and were apparently mitotically competent, as evidenced by the recovery of large clones consisting of more than 25 cells (Fig. 2D). This result provides evidence that even the complete lack of Cdk12 does not compromise cell viability, thus reinforcing the idea that Cdk12 is not a generally essential Pol II kinase and is not required for the transcription of essential and housekeeping genes at least in adult tissues.

### Cdk12 is critical for the expression of endogenous Nrf2 target genes

**Figure 3:**
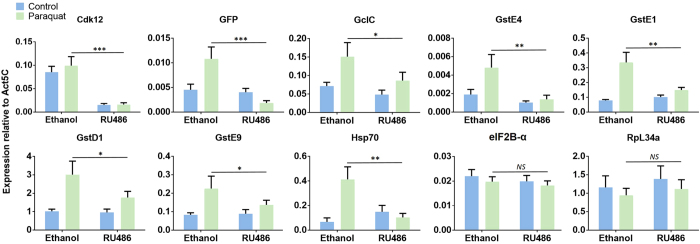
Cdk12 supports the expression of endogenous CncC target genes. RT-qPCR analyses showing that RU486-triggered ubiquitous Cdk12 knockdown reduced the upregulation of the mRNA levels of endogenous CncC target genes as well as the ARE-GFP reporter in response to the oxidative stressor paraquat. A known Cdk12-dependent gene, Hsp70, also showed decreased induction by paraquat in a Cdk12 knockdown background. RU486-induced ubiquitous Cdk12 knockdown in flies did not affect the mRNA expression levels of housekeeping genes eIF2B-α and Rpl34a in the presence or absence of oxidative stressor paraquat. Relative expression levels of individual genes were normalized to the internal control, Act5C. P values were calculated using multiple t-tests; *P < 0.05, **P < 0.005, ***P < 0.0005. Error bars represent standard deviations of 4 biological replicates.

Nrf2 controls gene expression programs that are critical for an organism’s ability to cope with oxidative stress. Prototypical Nrf2 target genes encode, for example, glutamate-cysteine ligases and glutathione S-transferases^35^. Glutamate-cysteine ligases are rate limiting enzymes in the synthesis of glutathione, a key antioxidant in maintaining the intracellular redox balance^36^. Glutathione S-transferases have critical functions in detoxification processes^37^,^38^. If Cdk12 were a positive regulator of Nrf2 target gene activity, we would expect the inducible expression of these genes to be compromised in Cdk12 loss-of-function conditions. To test this prediction, we exposed adult *Drosophila* to paraquat, a compound that stimulates the generation of superoxide radicals and can be used to elicit oxidative stress response in model organisms^39^,^40^. Paraquat-containing sucrose solution was fed to flies that carried a tubulin-GeneSwitchGal4 driver, a UAS-Cdk12^RNAi^ construct and an ARE-GFP reporter. RU486 was used to induce the expression of UAS-Cdk12^RNAi^ construct and then CncC target gene expression was quantified by RT-qPCR. In the absence of UAS-Cdk12^RNAi^ expression, paraquat treatment resulted in a substantial increase in the transcription level of Nrf2 target genes like GclC, GstD1 and GstE1; however, these genes lost up to 78% of their paraquat-induced expression when Cdk12 was knocked down, suggesting that the kinase contributes to the transcription regulation of endogenous CncC targets in response to oxidative stress (Fig. 3). A previously published Cdk12-dependent locus, the stress responsive Hsp70 gene^13^, also displayed robust induction upon paraquat administration, which was abrogated after Cdk12 knockdown. Despite the strong effect of Cdk12 knockdown on stress response genes, Cdk12 depletion did not affect the expression of two housekeeping genes we also tested here, eIF2B-α and RpL34a (Fig. 3). This finding confirms that Cdk12 is not required for the expression of every Pol II-transcribed gene and instead functions in a gene-selective manner.

Similar to what was observed with paraquat treatment, exposure to oltipraz, which activates the Nrf2 pathway through a different mechanism, significantly upregulated endogenous Nrf2 target genes in control flies; yet, after RU486-triggered Cdk12 knockdown, such increases were strongly diminished (Supplementary Fig. S3). Together with the previous experiment using paraquat treatment, these results confirmed that Cdk12 plays a critical role in mediating Nrf2-dependent target gene activation.

### Cdk12 promotes stress resistance and overall survival in oxidative stress conditions

**Figure 4:**
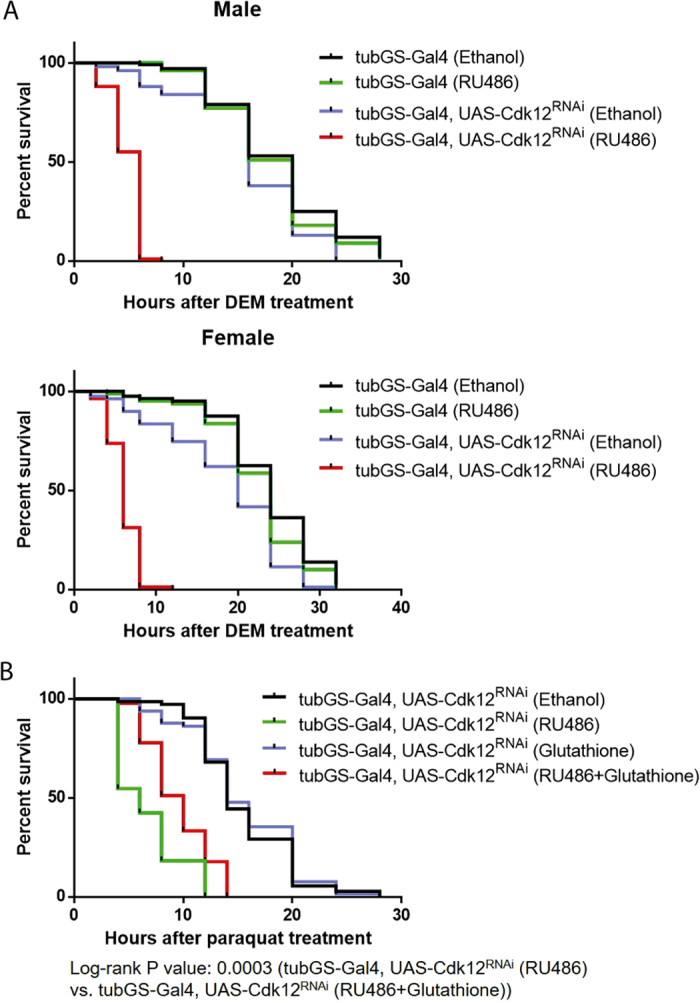
Cdk12 knockdown increases sensitivity to oxidative stress. (**A**) Survival curves for both male and female flies upon DEM exposure are shown. (**B**) Glutathione partially rescued the increased stress sensitivity caused by Cdk12 knockdown. The effect of glutathione supplementation was calculated using log-rank test by comparing the survival curves of tubGS-Gal4, UAS-Cdk12^RNAi^ (RU486) and tubGS-Gal4, UAS-Cdk12^RNAi^ (RU486 + glutathione). Glutathione addition had no effect on the survival of control flies with wild type levels of Cdk12. Male flies were used for this analysis.

The data presented above established Cdk12 as a positive regulator of CncC target genes. To evaluate the physiological relevance of this function, we assessed how Cdk12 might affect the stress sensitivity of flies. First, groups of flies with or without an RU486-inducible Cdk12^RNAi^ construct were fed with control or RU486-containing food. These flies were then exposed to a lethal dose of the oxidative stressor DEM and the time-course of survival was recorded. In line with our molecular data, both male and female flies with Cdk12 knockdown died much more quickly than control flies, demonstrating increased sensitivity to oxidative stress (Fig. 4A). Similarly, flies in which Cdk12 was knocked down showed increased sensitivity to paraquat, another oxidative stressor (Fig. 4B). Interestingly, the decline in survival caused by Cdk12 knockdown could partially be rescued by a dietary supplement of glutathione (Fig. 4B). The latter result argues that the increased lethality caused by Cdk12 depletion is, at least in part, due to a defect in glutathione synthesis, where CncC target genes play an important role. Therefore, it is plausible to conclude that the loss of CncC-regulated transcription in a Cdk12 loss-of-function background would result in reduced antioxidant capacity causing stress sensitivity.

### RNA-seq data suggest that Cdk12 may have opposing effects on oxidative stress response genes and housekeeping genes that control metabolic activities

Using reporter assays and RT-qPCR analyses, the previous experiments suggested that Cdk12 plays a key role in the protective responses to exogenous stressors including DEM and paraquat. To understand the effect and target selectivity of Cdk12 in stress-exposed flies on a genome-wide scale, we conducted RNA-seq experiments. Total RNA from flies that were subjected to Cdk12 knockdown, paraquat exposure, a combination of these two treatments, or no treatment, was then extracted and sequenced.

**Figure 5:**
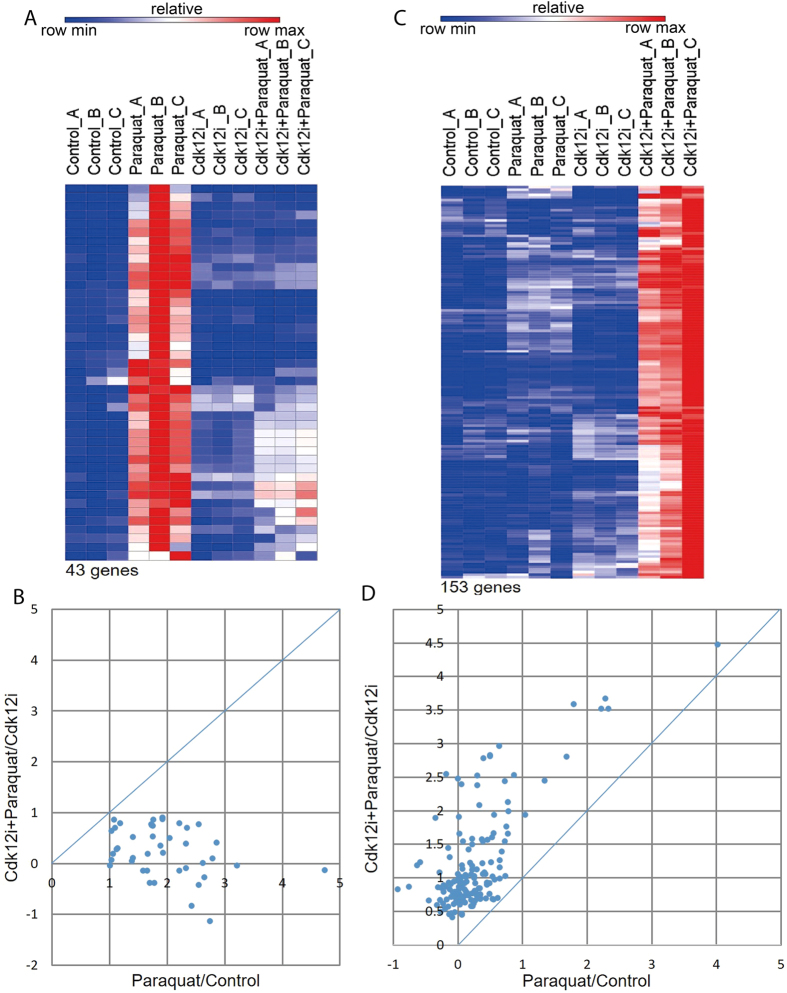
RNA-seq analysis identifies two distinct groups of genes affected by Cdk12 loss of function. (**A**) Heatmap of 43 genes that were strongly upregulated by paraquat exposure of adult flies in a Cdk12-dependent manner (See Supplementary Table S3 for the list of genes). (**B**) Scatterplot showing that under Cdk12 knockdown conditions, the genes described in Fig. 5A display lower fold induction by paraquat than under control conditions. (**C**) Heatmap of 153 genes which are Cdk12-dependently downregulated in paraquat-exposed flies (See Supplementary Table S4 for the list of genes). (**D**) Scatterplot showing that under Cdk12 knockdown conditions, the genes described in Fig. 5C display higher fold induction by paraquat than under control conditions.

Analysis of the RNA-seq data identified a group of genes that were significantly more abundant in the samples from paraquat-treated vs. control flies, and where this increase was abolished by Cdk12 knockdown. 43 genes were found in this group (Fig. 5A, the criteria used to identify this group are detailed in the Methods section). Some of the genes identified here, such as the GstE family genes, were also confirmed by qPCR results shown in Fig. 3. The scatterplot in Fig. 5B visualizes how Cdk12 knockdown diminished the fold induction of these genes. Gene ontology analysis revealed that the 43 genes are significantly enriched for glutathione transferase activity, peroxiredoxin activity, antioxidant activity and oxidoreductase activity, etc., suggesting that genes which require Cdk12 for paraquat-mediated expression are predominantly oxidative stress response genes (Table 2).

We also noticed a group of 153 genes for which Cdk12 appeared to have a rather distinct function. This group encompassed genes that were significantly upregulated by paraquat in Cdk12 knockdown conditions (Fig. 5C). Fold induction of these genes by paraquat in the absence or presence of Cdk12 RNAi was also shown in a scatterplot (Fig. 5D). As illustrated, these genes were more readily upregulated by paraquat in flies depleted of Cdk12, while in the presence of Cdk12, the effect of paraquat was minimized. This pattern suggests that when confronted with oxidative stress, Cdk12 functions to inhibit these genes. Gene ontology analysis revealed that these genes were significantly enriched for loci required for a variety of metabolic processes, such as the synthesis of amino acids, lipids and carbohydrates, et cetera (Table 3). This result might be biologically explained by assuming that for cells that are exposed to potentially fatal oxidative stress, it is beneficial to dial down metabolic rate in order to concentrate on stress defence and damage repair^41^. Specifically, in the presence of wild type levels of Cdk12, expression of metabolic genes is suppressed when exposed to a potentially lethal dose of oxidative stress; however, without Cdk12, such a control system becomes dysfunctional and their expression goes up. Therefore, a factor like Cdk12 might prevent or delay the upregulation of metabolic genes during stress exposure. Taken together, our RNA-seq data suggest that Cdk12 may be selectively required for the expression of stress response genes. The RNA-seq data further indicate that Cdk12 has a negative effect on genes that are involved in metabolic processes. Considering that the function of CTD kinases is to support transcription and RNA maturation, this repressive effect on metabolic genes is likely to be indirect. Thus, Cdk12 could act as a gene-selective Pol II kinase that directs differential gene expression on a genome-wide scale and contributes to the switch of cells from a functionally normal state to a “stress defence mode”, which is characterized by the upregulation of heat shock, oxidative stress and DNA damage response genes with the concomitant suppression of metabolic gene expression.

## Discussion

Here we present the Pol II CTD kinase CDK12 as a novel factor that is required for the function of CncC, the *Drosophila* homolog of the Nrf2 transcription factor. Even though it is assumed that the phosphorylation of Ser2 in the largest Pol II subunit is universally required for the transcription of protein-coding genes^27^,^32^, in our system we found that the function of Cdk12 is gene-selective rather than essential, and that it is needed for the inducible expression of oxidative stress response genes without affecting cell viability or basic housekeeping processes. This might be explained by assuming that the activity of the related kinase CDK9 supports the transcription of most genes^42^,^43^, but is not sufficient for the stress response genes including those regulated by CncC. The existence of gene-selective CTD kinases is not unique to *Drosophila*. Multiple CTD Ser2 kinases have been identified in other organisms as well. For example, the *S. cerevisae* genome encodes Bur1 (Cdk9 homolog) and Ctk1 (Cdk12 homolog). Notably, *ctk1* null mutants show cold sensitive phenotypes but are otherwise viable and healthy^44^,^45^, which correlates with our findings. In mammals CDK9, CDK12 and CDK13 are all CTD kinases with substrate specificity for serine 2^44^.

A gene selective function of Cdk12 has been recently discovered for certain classes of stress responsive transcription units. It was found that many genes that are involved in DNA repair, as well as heat shock genes require Cdk12 function in order to be transcriptionally activated in response to genotoxic or thermal stress, respectively^13^,^14^. Our results add to this picture by showing that Cdk12 also supports the oxidative stress response mediated by Nrf2. The addition of Nrf2 target genes to the group of Cdk12-dependent loci expands the known regulatory scope of the kinase. It also brings its specific role in the cellular response to stress, be it temperature, oxidative or genotoxic into focus. Notably, both qPCR and RNA-seq results indicate that the expression of Cdk12 itself is not elevated in response to oxidative stress. It is therefore unlikely that the upregulation of stress response genes is mediated or facilitated by an increase in Cdk12 synthesis at least at the transcription level.

The transcription and transcriptome studies presented above further confirm that Cdk12 is not essential for bulk RNA polymerase II transcription and that the majority of transcribed genes, including typical housekeeping genes, are not affected by loss of Cdk12 function. It is reasonable to assume that the related enzyme CDK9 mediates the phosphorylation of serine 2 residues for these genes, which aligns with published findings showing that compromised CDK9 function leads to inhibition of transcription of most Pol II molecules, severe growth defects and/or cell lethality^42^,^43^,^44^,^46^,^47^,^48^. Consistent with this interpretation, we found that whereas dsRNA-mediated knockdown of Cdk12 had no discernible effect on the activity of the actin promoter driven renilla luciferase reporter or on cell viability, Cdk9 knockdown reduced the readout of the actin reporter and appeared to decrease cell viability.

Among the proteins identified in our RNAi screen as required for Nrf2 reporter gene activation, Cdk12 had the most potent effect. In cell culture experiments, analysis of somatic cell clones, and at the organism levels, we confirmed that Cdk12 is critical for Nrf2 reporter activity and the expression of endogenous CncC target genes. In contrast, the *in vivo* experiments assessing Nrf2 activity indicated that Cdk12 is chiefly required for the induction of target gene expression in response to stress or other Nrf2-activating signals. The basal level of Nrf2 target gene expression, however, is less sensitive to depletion of the kinase. It follows that the function of Cdk12 is mostly required for stress inducible activation of Nrf2 regulated genes rather than for their basal transcription. At first glance the cell culture experiments in S2 cells seem to be at odds with this conclusion, as in this system we see Cdk12 knockdown affecting both the basal and the inducible levels of target gene expression. This might however be explained by a permanent stress exposure of these cells under standard cell culture conditions (21% O_2_). These conditions are actually hyperoxic and it is likely what we consider to be a “basal level” of Nrf2 target gene expression in S2 cells, is inherently elevated even without exogenous challenge.

RNA-seq experiments were conducted to study the function of Cdk12 in the stress response of an intact adult organism. This comprehensive analysis identified a group of genes that require Cdk12 function for transcriptional upregulation in response to oxidative stress. Consistent with the identification of Cdk12 as a positive regulator of CncC target genes, this group prominently includes known CncC downstream targets such as glutathione-S-transferases. Analyses of Cdk12-dependent transcriptome changes further revealed that the kinase suppressed the expression of a group of genes that are associated with metabolic functions in paraquat treated conditions. This group includes loci encoding enzymes that are involved in the biosynthesis of amino acids, nucleosides and other biomolecules. The opposite action of Cdk12 on antioxidant response and metabolic genes can potentially contribute to the switch between normal function and the “defence mode” as a strategy to survive adverse conditions and minimize damage. Specifically, these anabolic genes were upregulated by paraquat in Cdk12 loss-of-function conditions, whereas this upregulation was inhibited when Cdk12 functioned normally. The fact that these anabolic genes have a tendency to be upregulated in stress conditions might be biologically explained by assuming that once the stress exposure is over, the expression of these genes needs to be elevated in order to rebuild cellular components and replace damaged biomolecules; however, while stress persists, the most pressing need in cells is to activate genes that can directly counteract stress until it passes. Therefore, cells may upregulate different groups of genes during and after stress exposure, and Cdk12 might be one of the factors that coordinate gene transcription throughout these different stages.

It is generally believed that the gene-specific regulation of transcription is primarily controlled by promoter- or enhancer-binding transcription factors^49^,^50^. Our study, together with previous reports on the requirement of Cdk12 for heat shock and DNA damage responses, suggests that gene-selective transcription regulation can also occur at the level of Pol II CTD phosphorylation^13^,^14^,^30^,^51^. Our data suggest that Cdk12 favours the transcription of genes that are required when cells are in distress, which could result in a global reprogramming of gene expression and allow cells to transition into a “stress defence mode”. Thus, the function of different CTD kinases might be conceptually compared to bacterial sigma factors, which also interact directly with the transcription machinery to bring about global gene expression changes to adapt to stressful (temperature, starvation) or metabolic needs (nitrogen, iron limitation)^52^,^53^,^54^. It is also worth pointing out that human CDK12 has been found to be associated with RNA-processing factors and is involved in RNA processing^29^,^55^,^56^. It is possible that besides regulating gene expression at the level of transcription, CDK12 may also affect the expression of its target genes through its ability to modulate RNA processing. Future experiments will have to determine whether the stress-related functions of Cdk12 are mediated by its gene-specific effects on transcription elongation, RNA processing, or other functions.

Considering that different CTD kinases with the same substrate specificity are present in cells, it is conceivable that they serve as large-scale switches in genetic programming and facilitate the transition from a metabolically active cell to a cell that is in damage control and survival mode after receiving a potentially harmful oxidative, temperature, or genotoxic insult. More studies are warranted to elucidate the mechanisms by which different CTD kinases are engaged under different conditions, their target specificity and how they complement each other to fulfil the transcription needs of an organism.

## Methods

### RNAi screening

A panel of 420 kinases was knocked down in *Drosophila* Schneider 2 (S2) cells with 3 different dsRNA targeting each locus. The screen was carried out by Dr. Michael Boutros’ group in German Cancer Research Centre in a 384-well format. On day 1, 15,000 cells were added to each well, which was pre-loaded with 250 ng dsRNA. After 45 min starvation in serum free media, 20 μl of complete media was added to each well. On day 2, reporter plasmids ARE-Fluc and Act5C-Rluc were transfected following the protocol of the QIAGEN “Effectene Transfection Reagent”. For the following 3 days, plates were incubated at 25 °C. On day 6, the reporter read-out was collected. A complete list of the kinases targeted and their readout are available in Supplementary Table S1.

### Drosophila S2 cell culture

Drosophila S2 cells were maintained at a 25 °C incubator according to the S2-DRSC protocol provided by the Drosophila RNAi Screening Centre. The complete media contains Sigma-Aldrich “Shields and Sang M3 Insect Medium” (Product #3652), 10% Fetal Bovine Serum (FBS) and 1% *Penicillin*-*Streptomycin* solution.

### Plasmids and fly lines

Firefly luciferase reporter plasmids and the Act5C-driven renilla luciferase reporter plasmid were generated or acquired as described earlier^23^. Transgenic fly lines carrying an *in vivo* ARE-green or ARE-red reporter were generated in the lab as described earlier^23^. The UAS-Cdk12^RNAi^ fly line (stock number v25508) was obtained from the Vienna Drosophila Stock Centre. The TRiP-Cdk12^RNAi^ fly line (stock number 34838) was obtained from the Bloomington Drosophila Stock Centre. The fly lines used to generate Cdk12 mutant clones were obtained from the Bloomington Drosophila Stock Centre and listed as follows: Cdk12 mutant line carrying the Df(3L)Exel9065 allele (stock number 7949), hsFlp;;Dr/TM3 (stock number 26902), FRT80B (stock number 1988), and ubi-GFP, FRT80B (stock number 1620).

### dsRNA treatment of Drosophila S2 cells

The sequence of Cdk12 dsRNA primers (Forward: AGTGTGCAGCACTCACGAAG Reverse: TTACCAGCTCGGCAAATAGG) was acquired from the Genomic RNAi data base (http://www.genomernai.org/GenomeRNAi/f). The sequence of the GFP dsRNA primers was previously decribed^23^. A T7 promoter sequence (TAATACGACTCACTATAGG) was added to the 5′ end of each primer. dsRNA was synthesized using the Promega “T7 RiboMAX Express RNAi System” by amplifying from *Drosophila* genomic DNA or the pGreen H-Pelican plasmid^23^ to generate Cdk12 and GFP dsRNA, respectively. dsRNA was purified using the QIAGEN “RNeasy Mini Kit”. For dsRNA treatment of S2 cells, 5 × 10^6^ cells in 1 ml FBS-free media were plated in each well of a 6-well plate. 32 μg dsRNA was added to each well and incubated for an hour at 25 °C. 1 ml of FBS-containing complete media was then added to each well and the cells were incubated for 96 hours before harvesting or transfection.

### S2 cells transfection and luciferase assays

S2 cells were transfected with the ARE-Fluc and the Act5C-Rluc reporter plasmids using the calcium phosphate method. The methods for conducting transfection and luciferase assays were described earlier^23^.

### Drug treatment of S2 cells

Following transfection of S2 cells with luciferase reporters, 25 μM oltipraz (LKT Laboratories, Inc) or 100 μM DEM (Sigma-Aldrich) was added to S2 cell culture. For those treatments, 1000 × stock oltipraz or DEM solutions were prepared in DMSO. Stock solutions were first diluted 1:200 in S2 cell culture media, and then added 1:5 into S2 cells. Treated S2 cells were then incubated at 25 °C for 24 hrs.

### Drug treatment of flies

For RU486 treatment, 250 μl 80% ethanol ± 5 mM RU486 (Cayman Chemical Company) was added to the surface of the fly food and left to dry overnight in a chemical hood. 20 flies were then added to each vial and fed on RU486 food for 6 days by switching to new RU486 food every 2 days. For oltipraz treatment, flies were kept on food supplemented with 1 mM oltipraz for 72 hours before microscopy or RNA extraction. For paraquat treatment, flies were starved for 2 hours and then kept for 12 hours in vials containing a piece of filter paper, which was soaked with 5% sucrose ±25 mM Paraquat (Sigma-Aldrich).

### Microscopy

For visualization of GFP reporter activity, adult flies were placed on an apple juice plate and pictures were taken using a Leica MZ16 F fluorescence stereomicroscope. For imaging of ejaculatory bulb clones, ejaculatory bulbs were dissected and mounted using MOWIOL-DABCO media. Confocal pictures were then taken using a Leica TCS SP5 system.

### Clone generation in ejaculatory bulbs

Flies and crosses are kept at 18 °C except during heat shock. For Cdk12 knockdown clones, hsFlp; ARE-GFP, Act5C > STOP > Gal4, UAS-RFP/Cyo virgins were crossed with W1118 male or UAS-Cdk12^RNAi^ male flies in a vial and allowed to lay eggs for 48 hours. 96 hours after the flies were removed from the vial, the vial was incubated at 37 °C for 30 min. After adult flies emerged, flies that were both GFP and RFP positive were selected for dissection. For Cdk12 mutant clones, hsFlp ;; ubi-GFP, FRT80B/TM3 virgins were mixed with ARE-RFP/Cyo; *cdk12*^*Exel9065*^, FRT80B/TM3 male flies and allowed to lay eggs for 48 hours. 96–120 hours after the flies were removed from the vial, the vial was incubated at 37 °C for 1 hour. After adult flies emerged, flies that were both GFP and RFP positive were selected for dissection.

### RNA extraction and RT-qPCR analyses

All RT-qPCR experiments were carried out in 3 or 4 biological replicates. For each condition, cohorts of 25 female flies each were placed in separate vials. After drug treatments, 10 flies from each vial were subjected to RNA extraction using the TRIzol^®^ reagent. cDNA was generated using the Fermentas “Maxima Reverse Transcriptase”. cDNA was diluted 50 fold and used directly as the template for qPCR reactions. Each qPCR reaction was composed of 10 μl diluted cDNA, 1.25 μl 5 μM forward primer, 1.25 μl 5 μM reverse primer, and 12.5 μl Bio-Rad “iTaq Universal SYBR Green Supermix”. The primer sequences for each gene tested are listed in Supplementary Table S2. The reactions were run on a Bio-Rad MyiQ2 Two-Color Real-Time PCR Detection System.

### Stress sensitivity assay

For the stress sensitivity experiment shown in Fig. 4A, 2-day old flies of the indicated genotypes were kept on ethanol or RU486 food to induce Cdk12 RNAi expression. The surface of the food was soaked with ethanol or RU486 solution as described above. The flies were then starved for 2 hours prior to exposure to 20 mM DEM. The solvent for DEM was 5% sucrose solution with 10% ethanol. A piece of filter paper was soaked with the DEM/sucrose solution and placed in a vial containing 25 flies. A starting population of 100 flies was used for each condition and the number of flies surviving at every indicated time point was recorded. For the stress sensitivity assay shown in Fig. 4B, 2-day old female tubGS-Gal4, UAS-Cdk12^RNAi^ flies were kept on food containing different chemical combinations (+/−RU486, +/−glutathione) for 6 days. Stock RU486 solution in 80% ethanol and stock glutathione solution in water were first made and then dissolved in fly food with a final concentration of 320 μM RU486 and 200 mg/L L-glutathione reduced (Sigma-Aldrich). The food was changed every two days. The flies were then starved for 2 hours and exposed to a piece of filter paper soaked with 30 mM paraquat ±200mg/L glutathione in 5% sucrose solution. A starting population of 100 flies was used for each condition and the number of flies surviving at every indicated time point was recorded.

### RNA-seq experiment and sequencing data analyses

12 cohorts of tubGS-Gal4, UAS-Cdk12^RNAi^ flies were kept in separate vials. Triplicates of each of 4 conditions were treated as indicated (+/−paraquat, +/−Cdk12 RNAi) using protocol described in section “Drug treatment of flies”. After treatment 10 flies from each cohort were subjected to RNA extraction and 500 ng RNA from each sample was submitted to the UR Genomics Research Centre for cDNA library construction and sequencing. RNA concentration was determined with the NanopDrop 1000 spectrophotometer and RNA quality was assessed with the Agilent Bioanalyzer. The Illumina “TruSeq RNAv2 Sample Preparation Kit” was used for next generation sequencing library construction per manufacturer’s protocols. Briefly, mRNA was purified from 200 ng total RNA with oligo-dT magnetic beads and fragmented. First-strand cDNA synthesis was performed with random hexamer priming followed by second-strand cDNA synthesis. End repair and 3′ adenylation was then performed on the double stranded cDNA. Illumina adaptors were ligated to both ends of the cDNA, purified by gel electrophoresis and amplified with PCR primers specific to the adaptor sequences to generate amplicons of approximately 200–500 bp in size. The amplified libraries were hybridized to the Illumina single end flow cell and amplified using the Illumina cBot. Single end libraries were sequenced on the Illumina HISeq2500, generating ~20–25 million single end 100 nt sequence reads per sample. Sequenced reads were cleaned according to a rigorous pre-processing workflow (Trimmomatic − 0.32) before mapping them to the *D. melanogaster* genome (ncbi-build5.3) with SHRiMP2.2.3 (http://compbio.cs.toronto.edu/shrimp/). Cufflinks2.0.2 was then used to perform differential expression analysis with an FDR cut-off of 0.05 (95% confidence interval). To select the group of genes whose paraquat-induced expression was dependent on Cdk12, the following cut-offs were used: 1) genes with at least one read in every sample 2) genes that belong to a defined cluster which displays a specific low-high-low-low expression pattern across Control, Paraquat, Cdk12i, and Cdk12i+ Paraquat conditions by using the clustering algorithm based on One Minus Pearson Correlation 3) log2 > 1, q < 0.05 (Paraquat/Control) 4) log2 >−1 (Cdk12i/Control) 5) Mean (Cdk12i) < (Mean (Control) * Mean (Paraquat)) ^ 0.5. To select the group of genes whose expression was suppressed by Cdk12 in paraquat treated conditions, the following cut-offs were used: 1) genes with at least one read in every sample 2) genes that belong to a defined cluster which displays a specific low-low-low-high expression pattern across Control, Paraquat, Cdk12i, and Cdk12i + Paraquat conditions by using the clustering algorithm based on One Minus Pearson Correlation 3) log2 > 0, q < 0.05 (Cdl12i+ Paraquat/Paraquat) 4) q < 0.05 (Cdk12i/Control) 5) log2 > 0, q < 0.05, (Cdk12i+ Paraquat/Cdk12i) With selected groups of genes, their individual values were represented in heat maps using software GENE-E developed by Broad Institute. The gene ontology analyses were done using GENERIC GENE ONTOLOGY (GO) TERM FINDER, developed by the Lewis-Sigler Institute at Princeton.

## Additional Information

**How to cite this article**: Li, X. *et al.* Cdk12 Is A Gene-Selective RNA Polymerase II Kinase That Regulates a Subset of the Transcriptome, Including Nrf2 Target Genes. *Sci. Rep.* **6**, 21455; doi: 10.1038/srep21455 (2016).

## Supplementary Material

Supplementary Information

Supplementary Table S1

Supplementary Table S2

Supplementary Table S3

## Figures and Tables

**Table 1:** RNAi screen identified a panel of kinases that potentially regulate the Nrf2 signalling pathway.

	Gene	RNAi 1	RNAi 2	RNAi 3
Control	CncC	−4.52		
	MafS	−3.61		
	Keap1	0.7		
Inducers	Cdk12	−25.1	−28.27	−39.64
	fray	−3.4	−3.82	−0.19
	Madm	−1.92	−2.86	−3.29
	Psi	−0.93	−2.84	−3.02
	CK2α	−1.93	−1.94	−1.95
Suppressors	Fs(1)h	1.96	1.9	1.6
	GSK-3	2.09	1.88	1.19
	Nipped-A	1.27	1.81	1.81

**Table 2:** Gene ontology analysis of Cdk12-dependent genes in paraquat response.

Gene Ontology term	Clusterfrequency	Genomefrequency	CorrectedP-value
glutathione transferase activity	14.30%	0.30%	4.22E-08
peroxiredoxin activity	7.10%	0.10%	6.32E-05
antioxidant activity	9.50%	0.20%	0.00012
oxidoreductase activity, acting on peroxide as acceptor	7.10%	0.20%	0.00189
glutathione peroxidase activity	4.80%	0.00%	0.00848
thioredoxin peroxidase activity	4.80%	0.00%	0.00848
oxidoreductase activity	19.00%	4.00%	0.01037

**Table 3:** Gene ontology analysis of genes that are suppressed by Cdk12 in paraquat treated conditions.

Gene Ontology term	Clusterfrequency	Genomefrequency	CorrectedP-value
single-organism metabolic process	41.4%	13.2%	2.69E-15
small molecule metabolic process	23.7%	4.7%	2.84E-13
carboxylic acid metabolic process	16.4%	2.1%	1.06E-12
organic acid metabolic process	16.4%	2.2%	3.30E-12
oxoacid metabolic process	16.4%	2.2%	3.30E-12
cellular amino acid biosynthetic process	6.6%	0.2%	8.57E-10
alpha-amino acid metabolic process	8.6%	0.6%	9.41E-09
alpha-amino acid biosynthetic process	5.9%	0.2%	1.47E-08
cellular amino acid metabolic process	9.9%	1.0%	4.31E-08
single-organism biosynthetic process	16.4%	3.5%	7.85E-08
organic acid biosynthetic process	7.2%	0.5%	1.41E-07
carboxylic acid biosynthetic process	7.2%	0.5%	1.41E-07
catabolic process	17.8%	4.4%	3.60E-07
small molecule biosynthetic process	7.9%	0.8%	1.67E-06
metabolic process	61.2%	37.6%	2.10E-06
single-organism catabolic process	10.5%	1.9%	2.64E-05
lipid metabolic process	11.2%	2.5%	0.00015
organic substance catabolic process	13.2%	3.6%	0.00035
cellular metabolic process	48.7%	29.7%	0.00041
organonitrogen compound metabolic process	23.0%	9.5%	0.00041
