# Supplementary material for: Cdk12 Is A Gene-Selective RNA Polymerase II Kinase That Regulates a Subset of the Transcriptome, Including Nrf2 Target Genes
Source: Sci Rep. 2016 Feb 25;6:21455. doi: 10.1038/srep21455 (PMC4766476; doi:10.1038/srep21455)
Supplement: Supplementary Information [file srep21455-s1.pdf]

Cdk12 Is A Gene-Selective RNA Polymerase II Kinase That Regulates a Subset of the Transcriptome, Including Nrf2 Target Genes

Xuan Li, Nirmalya Chatterjee, Kerstin Spirohn, Michael Boutros & Dirk Bohmann

Supplementary Information

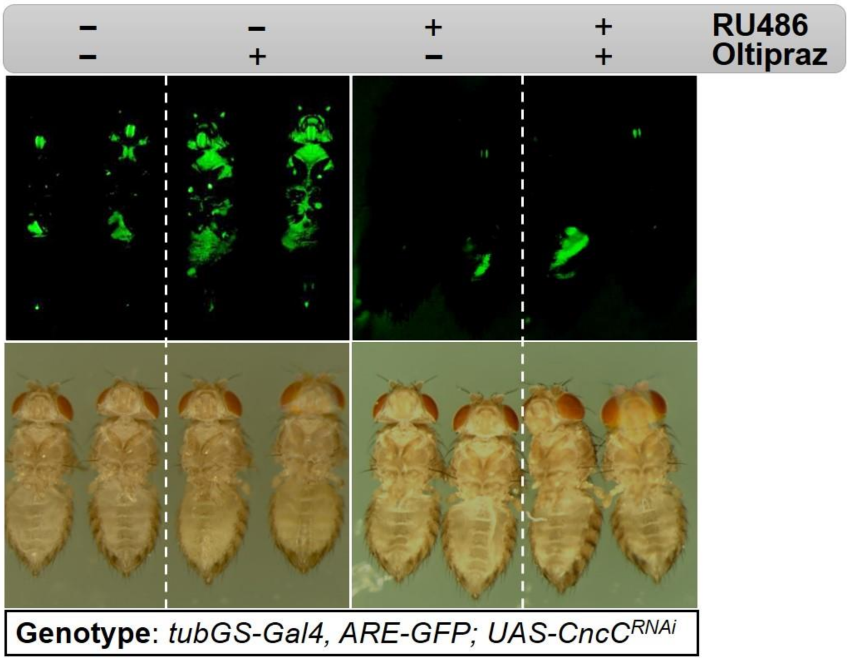

Supplementary Figure S1. Nrf2 reporter activation by oltipraz is suppressed in CncC knockdown flies. Knockdown of CncC by expression of an RNAi construct under the control of the ubiquitous gene-switch tub-Gal4 driver inhibits oltipraz-induced ARE-GFP reporter activation in adult flies. The upper and lower panels show GFP fluorescence and bright field images, respectively, of the same flies. Two females are shown for each condition.

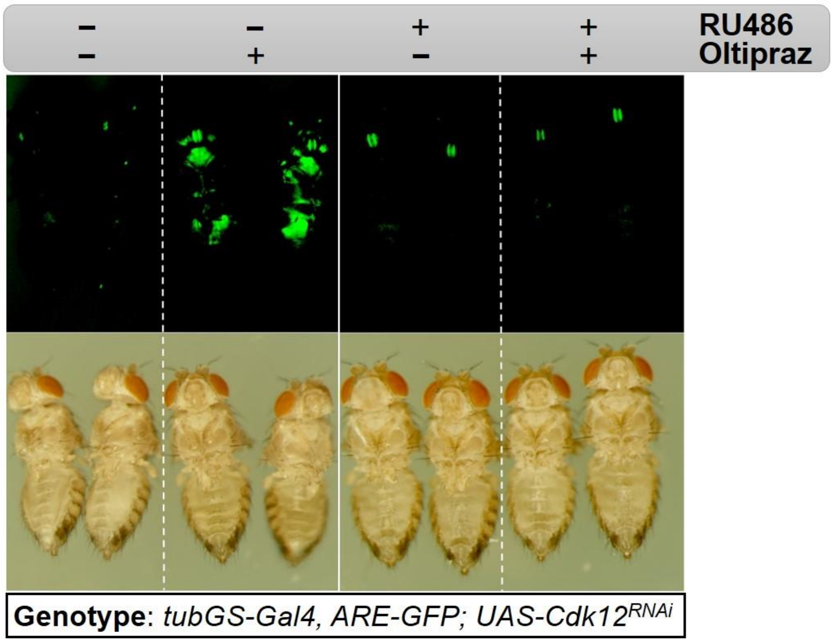

Supplementary Figure S2. Cdk12 is required for the optimal induction of Nrf2 reporter activity. The result described in Fig. 2A was reproduced using an independent fly line that expresses a hairpin RNA targeted to a different, non-overlapping region of Cdk12 mRNA. The upper and lower panels show GFP fluorescence and bright field images, respectively, of the same flies. Two females are shown for each condition.

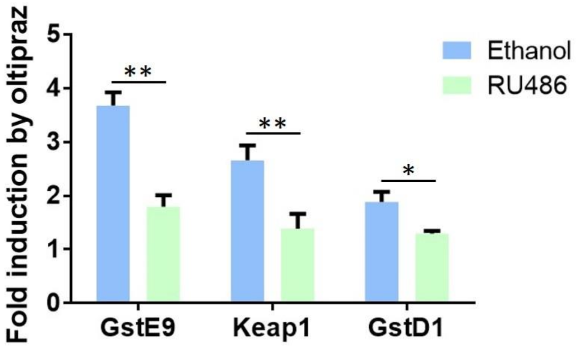

Supplementary Figure S3. Cdk12 supports the induced expression of endogenous Nrf2 target genes. Ubiquitous Cdk12 knockdown in flies reduced the fold induction of endogenous Nrf2 target genes in response to oltipraz. Relative expression levels of individual genes were normalized to the internal control, Act5C. P values were calculated using multiple t-tests; *P<0.05, ** P<0.005. Error bars represent standard deviations of 3 biological replicates.

Supplementary Table S2: Primer sequences for qPCR analyses

Gene Name	Forward Primer	Reverse Primer
**GFP**	AACCGCATCGAGCTGAAGG	TGGGTGCTCAGGTAGTGGTTG
**Cdk12**	CCCGACGAAATGCCAACGCC	CGACTCCTGTTGCTCCCGCA
**GclC**	CGCTTTATCCAGAAGCGTGCCG	AATCCTGCTTGTAATCCGGGTGGC
**GstE4**	TGCTGCAGCGTGCCAAAGTG	ACCGGACGGGTGAGTCTCCT
**GstE1**	GCTGGAGACGTTCCTGGGCAA	ACAGTGGGTCCGGTGGACAGA
**GstD1**	GGCCGCCTTCGAGTTCCTGA	CGGTTGCCACCAGGGCAATG
**GstE9**	GGTGCCGCGTTCCCAGATTG	GACCGGTCCGCAGAGGTAAGC
**Hsp70**	GGTCACATCGCCGGCCTGAAT	AGCACATTGCGCTCACCCTTGA
**eIF2B-α**	ATCAACCGCATTGGCACCTA	AAGCTTTCGGCCAGAACGTA
**RpL34a**	TCCAAGCGTTTGAAGACCGT	GGACGATTCTCTCTCGCAGG
